# 1,8-Cineole Alleviates Hippocampal Oxidative Stress in CUMS Mice via the PI3K/Akt/Nrf2 Pathway

**DOI:** 10.3390/nu17061027

**Published:** 2025-03-14

**Authors:** Wenze Wu, Dequan Wang, Yuzhu Shi, Yichen Wang, Yongzi Wu, Xinyan Wu, Basit Ali Shah, Gang Ye

**Affiliations:** 1 College of Life and Health Science, Northeastern University, Shenyang 110819, China; 2 College of Food Science and Nutritional Engineering, China Agricultural University, Beijing 100083, China; 3 School of Biomedical Engineering, Guangzhou Medical University, Guangzhou 511436, China; 4 College of Veterinary Medicine, Sichuan Agricultural University, Chengdu 625014, China

**Keywords:** 1,8-cineole, depression, functional foods, health supplement, Nrf2, oxidative stress

## Abstract

**Background**: This study investigates the neuroprotective effects of 1,8-cineole (1,8-CH), against hippocampal oxidative stress in a chronic unpredictable mild stress (CUMS) mice model of depression, focusing on the underlying molecular mechanisms. **Methods**: The effects of CUMS exposure were assessed by measuring oxidative stress markers, antioxidant activity, and neuronal damage in the hippocampus using histopathology, network pharmacology, Western blot analysis, and small interfering RNA (siRNA) knockdown experiments. **Results**: 1,8-CH significantly alleviated depression-like behaviors in CUMS mice. CUMS exposure induced oxidative stress in the hippocampus, evidenced by elevated MDA levels, decreased antioxidant activity, and neuronal damage. DHE staining revealed ROS accumulation. Treatment with 1,8-CH alleviated oxidative stress by reducing MDA, restoring antioxidant activity, and lowering ROS levels, while improving neuronal structure. Network pharmacology identified the PI3K/Akt/Nrf2 pathway as a key mediator of 1,8-CH’s neuroprotection, which was supported by Western blot results, demonstrating PI3K/Akt activation and a potential enhancement of Nrf2 nuclear translocation. Furthermore, in corticosterone-induced PC12 cells, the antioxidant effects of 1,8-CH were abolished by Nrf2 inhibition and siRNA knockdown, confirming Nrf2’s role. **Conclusions**: These findings suggest that 1,8-CH alleviates hippocampal oxidative stress in CUMS-induced depression via the PI3K/Akt/Nrf2 pathway, highlighting its potential as a health supplement for managing depression.

## 1. Introduction

Depression is a widespread and debilitating disorder that affects millions of individuals worldwide, significantly impairing daily functioning and overall quality of life [1,2]. It is often characterized by persistent sadness, anhedonia, and emotional numbness [3]. Despite its widespread impact, the severity of depression is often underestimated, contributing to disability and premature mortality. While traditional treatments, such as antidepressant medications and psychotherapy, are effective for some individuals, they may not be universally effective and can be associated with adverse side effects [4]. This has sparked increasing interest in dietary bioactive compounds as potential complementary or alternative treatments for depression. These compounds, present in foods such as fruits and vegetables, have been shown to exhibit neuroprotective and mood-enhancing effects [5,6]. Bioactive substances such as polyphenols, flavonoids, omega-3 fatty acids, and amino acids like tryptophan have shown promise in alleviating depressive symptoms [7,8,9]. These compounds exert their effects through mechanisms such as modulating inflammation, reducing oxidative stress, and regulating neurotransmitter activity. As research progresses, these compounds may provide natural and effective strategies for the management of depression and the enhancement of overall mental well-being.

The investigation of monoterpenoid compounds, such as α-pinene, β-caryophyllene, α-phellandrene, limonene, β-linalool, 1,8-cineole, β-pinene, caryophyllene oxide, p-cymene, and eugenol, presents significant potential in advancing antidepressant research [10]. These compounds exhibit a wide range of bioactivities, such as neuroprotection, anti-inflammatory effects, antibacterial properties, the modulation of neurotransmitter systems, and antioxidant actions [11]. Among them, 1,8-cineole (1,8-CH) has attracted particular attention due to its potential to alleviate depressive symptoms by enhancing serotonin and dopamine signaling, positioning it as a promising natural alternative for depression treatment [12,13]. 1,8-CH, also known as eucalyptol, is a functional dietary bioactive compound found in the essential oils of plants such as eucalyptus, rosemary, and sage [14,15]. Recent studies suggest that 1,8-CH may positively modulate neurotransmitter systems, particularly serotonin and dopamine, which are key regulators of mood [12,13,16]. Furthermore, 1,8-CH plays a critical role in reducing oxidative stress, a key contributor to depression and various neurodegenerative disorders [17,18]. Its antioxidant properties can alleviate the damaging effects of free radicals, which can impair brain function and exacerbate mood disturbances. In addition to its mood-enhancing effects, 1,8-CH also demonstrates mild anxiolytic and antidepressant-like actions, further supporting its potential as a valuable tool for managing both anxiety and depression [19]. Despite these promising findings, research specifically exploring the antidepressant effects of 1,8-CH remains limited. Therefore, further investigation is needed to fully understand its therapeutic potential.

Network pharmacology, a systems-based approach, offers valuable insights into the complex interactions between bioactive compounds and biological networks, specifically in the context of depression [20]. This approach can identify how functional dietary bioactive compounds such as polyphenols, flavonoids, and omega-3 fatty acids influence multiple molecular targets involved in depressive mechanisms. By elucidating the effects of these compounds on various biological pathways, network pharmacology provides their potential role as health supplements in modulating mood and supporting mental well-being. Molecular docking further enhances this understanding by predicting the binding interactions between these compounds and specific proteins, enzymes, or receptors associated with depressive disorders [21]. Collectively, these approaches offer a comprehensive framework for exploring how health supplements can contribute to mental health, mitigate the risk of depression, and support the development of targeted dietary interventions aimed at alleviating depressive symptoms.

This study employed a chronic unpredictable mild stress (CUMS) model to evaluate the antidepressant potential of 1,8-CH as a health supplement. To our knowledge, this is the first study to integrate network pharmacology for a comprehensive exploration of the molecular mechanisms and targets underlying 1,8-CH’s antidepressant effects. By identifying key biological pathways and molecular interactions, we aimed to elucidate the mood-regulating properties of 1,8-CH.

## 2. Materials and Methods

### 2.1. Chemicals and Reagents

1,8-CH was obtained from Chengdu Must Bio-Technology Co., Ltd. (Chengdu, China). Venlafaxine hydrochloride capsules (VLFs) were sourced from Chengdu Kanghong Pharmaceutical Group Co., Ltd. (Chengdu, China). Hematoxylin and eosin (HE) were provided by Solabio (Beijing, China). Malondialdehyde (MDA) and superoxide dismutase (SOD) assay kits were procured from Nanjing Jiancheng Bioengineering Institute, Nanjing, China. The following antibodies were utilized: PI3K (Bioworld), p-PI3K (Genetex, Irvine, CA, USA), AKT (Bioworld, Irving, TX, USA), p-AKT (CST, Danvers, MA, USA), HO-1 (CST, USA), Keap1 (Proteintech, Rosemont, IL, USA), Nrf2 (CST, USA), β-actin (CST, USA), and Histone H3 (Abcam, Cambridge, UK).

### 2.2. Animals

Thirty-six male C57BL/6 mice (22–25 g) were procured from Liaoning Changsheng Biotechnology Co., Ltd. (Shenyang, China). The animals were housed under controlled conditions in clean cages, with unrestricted access to both water and food. The environmental parameters were maintained at a temperature of 20–25 °C and a relative humidity of 50–60%, with daily exposure to adequate natural light.

### 2.3. CUMS Depression Model

After one week of acclimation to the controlled laboratory environment, the mice were randomly assigned to one of six groups (eight animals per group): the control group, the CUMS group, the positive control group (VLF, 10 mg/kg·d^−1^, administered orally), and the 50 mg/kg·d^−1^, 100 mg/kg·d^−1^, and 150 mg/kg·d^−1^ treatment groups (1,8-CH, administered orally).

The CUMS paradigm was carried out as outlined by Willner et al. [22], with slight modifications. In brief, with the exception of the control group, the mice in all experimental groups were exposed to one or two stressors daily. These stressors included the following: 12 h of fasting, day–night cycle inversion, a 45° tilt of the cage for 24 h, a wet cage environment for 24 h, exposure to 90 dB noise for 12 h, immersion in cold (4 °C) or hot (45 °C) water for 4 min, placement in an empty cage for 3 h, flashlight exposure (150 flashes/min) for 6 h, and restraint for 3 h. The stressors were administered in a randomized sequence, with no stressor repeated on consecutive days. All procedures were conducted in strict accordance with the 3R principles. These experiments were approved and conducted following the guidelines set by the Biological and Medical Ethics Committee of Northeastern University (approval number: NEU-EC-2024A181S), and complied with the ARRIVE guidelines.

### 2.4. Sucrose Preference Test (SPT) and Open-Field Test (OFT)

The mice were acclimated to a sugar-supplemented water solution for a duration of 1 h. Two water bottles were provided in each cage: one containing a 1% sucrose solution and the other with pure water. To mitigate potential side preference, the positions of the water bottles were alternated after 30 min. The consumption of both sucrose solution and pure water was subsequently recorded. Prior to the experiment, the mice were allowed ad libitum access to food, and the volumes of both the tap water and sucrose solution consumed were measured by weighing the bottles. Sugar water preference was calculated as the proportion of sugar water intake relative to the total fluid consumption, expressed as follows: sugar water preference (%) = (sucrose solution consumption (g)/[sucrose solution consumption (g) + water consumption (g)]) × 100%.

The OFT was performed in a cubic arena with dimensions of 50 cm × 50 cm × 50 cm. At the beginning of each trial, a mouse was placed in one of the arena’s corners, and its behavior was recorded for a duration of 5 min using an overhead camera. Following each trial, the arena was thoroughly disinfected with 75% ethanol to eliminate any potential contaminants. Subsequently, the Sassafras was carefully dried using paper towels to ensure the complete evaporation of any remaining moisture.

### 2.5. Forced Swimming Test (FST)

Mice were individually placed in a transparent Plexiglas cylinder (25 cm in height and 10 cm in diameter), filled with water to two-thirds of its height at 24 °C. Each trial lasted 5 min and was video-recorded. The duration of immobility was quantified by analyzing the video footage. Immobility was defined as the absence of voluntary movement, with the exception of those movements required for maintaining flotation.

### 2.6. HE Staining

Brain tissue sections were deparaffinized in xylene, rehydrated through a graded ethanol series, and stained with hematoxylin for 5–10 min to highlight the nuclei. The sections were then stained with eosin for 1–2 min to color the cytoplasm. Afterward, they were dehydrated, cleared in xylene, mounted with a coverslip, and examined under a light microscope for detailed histological analysis, allowing for the assessment of tissue morphology and structure.

### 2.7. Dihydroethyl Chloride (DHE) Assay

The production of reactive oxygen species (ROS) in 20 μm thick brain sections was quantified using DHE fluorescence staining. Tissue sections were incubated with 10 μM DHE in phosphate-buffered saline (PBS, pH 7.4) at 37 °C for 30 min in the dark, followed by three washes with PBS (5 min per wash) to remove excess dye. Samples were subsequently mounted for imaging using confocal laser scanning microscopy (Zeiss LSM 980, Zeiss, Jena, Germany) with excitation/emission wavelengths set at 518/616 nm and a 20× objective lens. Fluorescence images were acquired, and maximum intensity projections were analyzed using ImageJ (v1.53) with standardized thresholding parameters to ensure consistency in quantification.

### 2.8. Network Pharmacology

A network pharmacology approach was employed to explore the potential health benefits of 1,8-CH as a health supplement for alleviating depressive symptoms. The analysis began by identifying potential targets from comprehensive databases like a traditional Chinese medicine systems pharmacology (TCMSP) database (http://tcmspw.com/tcmsp.php, accessed on 2 January 2025), PubChem (https://pubchem.ncbi.nlm.nih.gov, accessed on 2 January 2025), and DrugBank (https://go.drugbank.com, accessed on 3 January 2025), which were then mapped to depression-related genes using resources such as GeneCards (https://www.genecards.org, accessed on 5 January 2025), DisGeNET (https://disgenet.com, accessed on 5 January 2025), and OMIM (https://www.omim.org, accessed on 5 January 2025). Protein–protein interaction (PPI) networks were constructed using the STRING database (https://cn.string-db.org, accessed on 8 January 2025), followed by functional enrichment analysis through DAVID and ClueGO. Gene Ontology (GO) and Kyoto Encyclopedia of Genes and Genomes (KEGG) pathway analyses further identified key biological processes and molecular mechanisms.

### 2.9. Molecular Docking

The three-dimensional structure of 1,8-CH was generated and energetically optimized using the LigPrep module (Schrödinger Suite, 2023-2) with the OPLS4 force field, applying a convergence threshold of 0.01 Å for bond geometry refinement. The crystal structure of the target protein was preprocessed using Schrödinger’s Protein Preparation Wizard (v13.0), which involved optimizing the hydrogen bond network, performing restrained minimization (RMSD cutoff: 0.3 Å), and assigning protonation states at physiological pH (7.4). A receptor grid (20 × 20 × 20 Å) centered on the canonical binding site was generated using the Receptor Grid Generation tool. Flexible docking simulations were conducted using Glide (SP mode) with 50 independent conformational sampling cycles. Binding poses were ranked according to GlideScore (ΔG, kcal/mol), and further validated through MM/GBSA energy calculations.

### 2.10. MDA and SOD Assays

The MDA and SOD assays were performed using commercially available kits, following the manufacturer’s instructions. For tissue samples, homogenates were prepared at 10% (*w*/*v*) in PBS and centrifuged at 12,000× *g* for 10 min at 4 °C, after which the supernatants were collected for analysis. For cultured cells, lysates were prepared using radioimmunoprecipitation assay (RIPA) buffer, incubated on ice for 30 min, and subsequently centrifuged at 12,000× *g* for 15 min. MDA levels were determined via the thiobarbituric acid (TBA) reaction, with absorbance measured at 532 nm, while SOD activity was quantified using WST-8-based assay kits at 450 nm. All measurements were normalized to protein concentration, determined using the bicinchoninic acid (BCA) assay, and expressed as U/mg protein.

### 2.11. Western Blot

Western blotting was performed by first extracting proteins from the samples and quantifying their concentrations using a protein assay. Equal amounts of protein were loaded onto an SDS-PAGE gel and separated by electrophoresis. The separated proteins were transferred onto a PVDF membrane, which was then blocked with 5% non-fat dry milk to prevent non-specific binding. The membrane was incubated with primary antibodies specific to the target protein, followed by HRP-conjugated secondary antibodies. Protein bands were detected using an enhanced chemiluminescence (ECL) detection system, and images were captured for analysis.

### 2.12. Cell Modeling and Treatment

PC12 cells were cultured until approximately 80% confluence was achieved. The culture medium was removed, and the control group was replenished with RPMI 1640 medium, while the model and treatment groups received RPMI 1640 medium supplemented with 250 μM corticosterone (CORT) for 24 h.

### 2.13. Cell Viability Assay

The cell viability was tested by 3-(4, 5-dimethylthiazol-2-yl)-2, 5-diphenyltetrazolium bromide (MTT) assay. The PC12 cells were seeded in 100 μL DMEM at 6000 cells/well in 96-well plates. The cells were incubated with MTT for 4 h at 37 °C, and DMSO was used to dissolve formazan. Finally, the optical density (OD) value was surveyed at an absorbance wavelength of 490 nm.

### 2.14. Small Interfering RNA (siRNA) Silencing

Following previous protocols [23], PC12 cells (1.5 × 10^5^ cells per well) were seeded in 24-well plates and transfected with either negative control siRNA or Nrf2-specific siRNA using a small-interfering RNA kit (GenePharma, Shanghai, China), according to the manufacturer’s instructions. Nrf2 siRNA was achieved from GenePharma (Shanghai, China). The siRNA sequences were as follows: sense, GAAUGGUCCUAAAACACCAtt; antisense, UGGUGUUUUAGGACCAUUCtg. After 60 h for protein expression analysis, silencing efficiency was assessed.

### 2.15. Statistical Analysis

Statistical analyses were performed using SPSS software (version 22.0; SPSS Inc., Chicago, IL, USA). Biochemical parameter differences between groups were evaluated using one-way analysis of variance (ANOVA). A *p*-value of less than 0.05 was considered indicative of statistical significance.

## 3. Results

### 3.1. 1,8-CH Significantly Mitigates Depressive-like Behaviors in CUMS-Induced Mice

**Figure 1:**
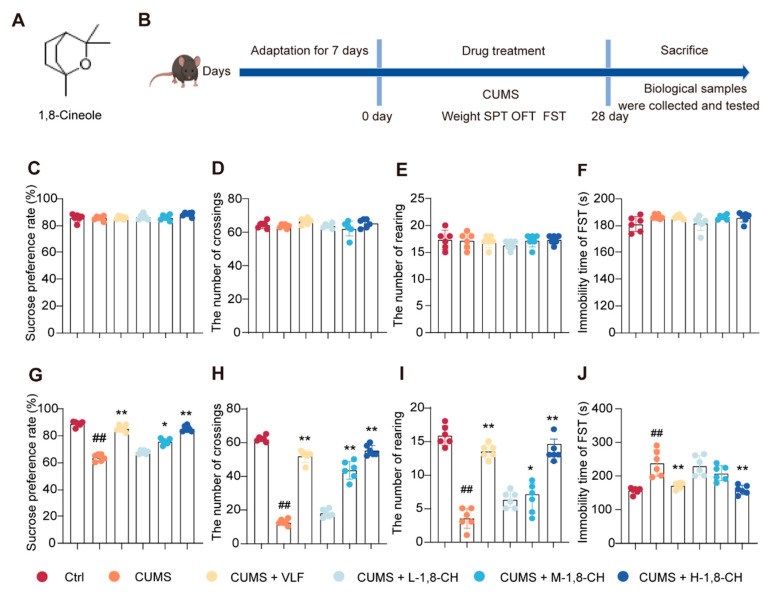
1,8-CH significantly mitigates depressive-like behaviors in CUMS-induced mice. (**A**) Chemical structure of 1,8-CH. (**B**) Schematic representation of the experimental protocol. (**C**–**F**) Sucrose preference rate, number of crossings in the OFT, number of rearing in the OFT, and immobility time in the FST on day 0. (**G**–**J**) Sucrose preference rate, number of crossings in the OFT, number of rearing in the OFT, and immobility time in the FST on day 28. L-CIN, M-CIN, and H-CIN correspond to low, medium, and high doses of 1,8-CH, respectively. Data are expressed as mean ± SD (n = 3). ^#^ *p* < 0.05, ^##^ *p* < 0.01 compared with control group; * *p* < 0.05, ** *p* < 0.01 compared with CUMS group.

In this study, the SPT, OFT, and FST were employed to assess depressive-like behaviors in mice (Figure 1A,B). As shown in Figure 1C–F, no significant differences were observed between the groups on day 0, indicating baseline equivalence. As anticipated, by the fourth week, CUMS significantly impaired the behavioral parameters, including a reduction in the sucrose preference index in the SPT, a decrease in rearing and crossing frequencies in the OFT, and an increase in immobility duration in the FST (Figure 1G–J). Notably, the administration of 1,8-CH for four weeks effectively mitigated the CUMS-induced behavioral abnormalities in a dose-dependent manner. These findings suggest that 1,8-CH can possess significant potential as a natural supplement with antidepressant-like effects.

### 3.2. 1,8-CH Alleviates Hippocampal Tissue Damage and Oxidative Stress in CUMS Mice

**Figure 2:**
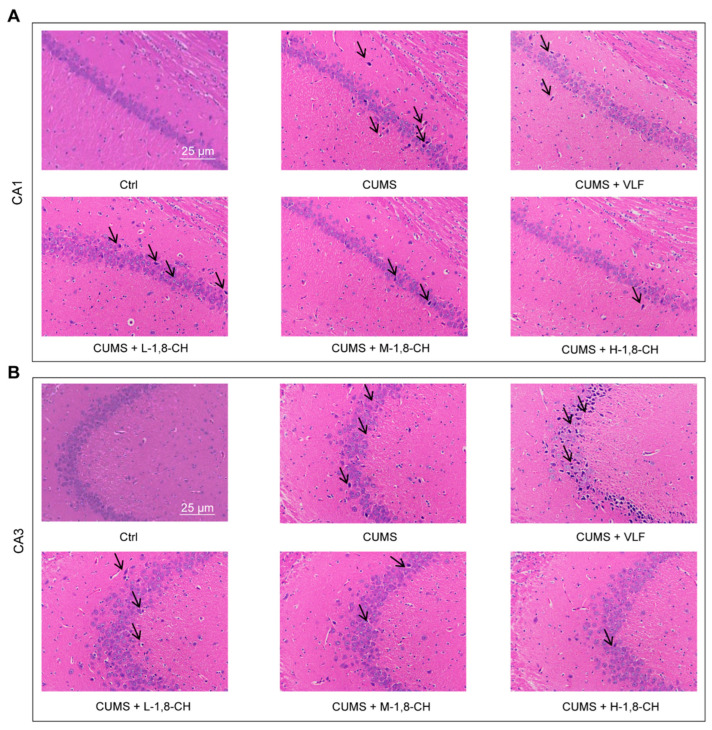
1,8-CH alleviates hippocampal tissue damage in CUMS mice. (**A**) Histopathological analysis of the CA1 region in hippocampal tissue of CUMS mice. (**B**) Histopathological analysis of the CA3 region in hippocampal tissue of CUMS mice. L-CIN, M-CIN, and H-CIN correspond to low, medium, and high doses of 1,8-CH, respectively. Black arrows denote representative pathological characteristics.

The hippocampus plays a critical role in the development of depression, as it is central to regulating emotional responses, learning, memory, and stress adaptation [24]. In this study, HE staining revealed significant hippocampal tissue abnormalities in mice subjected to CUMS (Figure 2A,B). The CUMS group showed reduced neuronal density, shrunken neurons, and disorganized tissue architecture within the hippocampus, indicative of neuronal degeneration and atrophy. In contrast, the 1,8-CH-treated group showed a marked improvement in hippocampal morphology, with restored neuronal density and a more organized tissue structure. Neurons in the 1,8-CH group appeared more robust, with well-defined cell bodies and less signs of degeneration compared to the CUMS group. These results suggest that 1,8-CH exerts a protective effect on hippocampal tissue.

**Figure 3:**
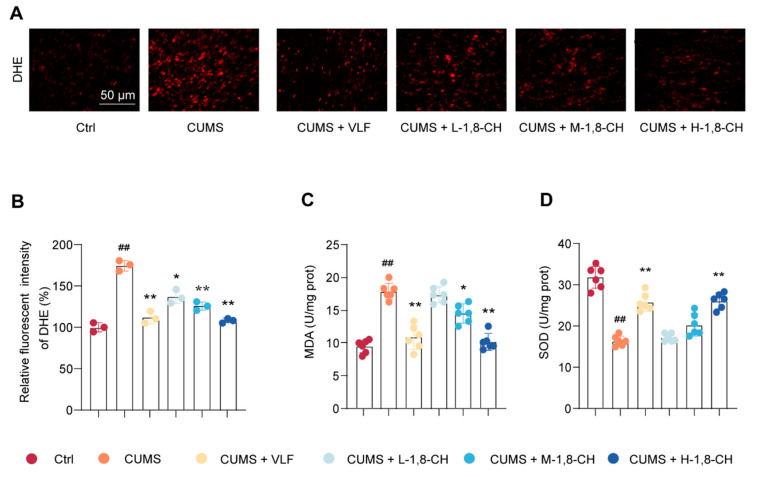
1,8-CH alleviates oxidative stress in the hippocampal tissue of CUMS mice. (**A**,**B**) Measurement of O^2−^ levels in hippocampal tissue using DHE staining. (**C**) Measurement of MDA levels using an assay kit. (**D**) Measurement of SOD levels using an assay kit. L-CIN, M-CIN, and H-CIN correspond to low, medium, and high doses of 1,8-CH, respectively. The sample size for DHE in each group was n = 3, while for other samples, it was n = 6. Data are expressed as mean ± SD. ^#^ *p* < 0.05, ^##^ *p* < 0.01 compared with control group; * *p* < 0.05, ** *p* < 0.01 compared with CUMS group.

In this study, the effects of 1,8-CH on oxidative stress markers in the hippocampus of mice exposed to CUMS were systematically evaluated. DHE staining, a well-established marker of reactive ROS, revealed a significant increase in oxidative stress within the hippocampus of CUMS-exposed mice, as evidenced by a prominent accumulation of red fluorescence (Figure 3A,B). Conversely, treatment with 1,8-CH resulted in a significant reduction in DHE staining, indicating a marked attenuation of ROS production in the hippocampus. To further assess the oxidative stress response, MDA levels, a key marker of lipid peroxidation, and SOD activity, an important antioxidant enzyme, were measured (Figure 3C,D). The CUMS group showed a significant elevation in MDA levels and a marked decrease in SOD activity. In contrast, 1,8-CH treatment significantly reduced MDA levels and increased SOD activity, effectively restoring the hippocampal antioxidant defense system. Collectively, these findings suggest that 1,8-CH exerts a protective effect against oxidative stress in the hippocampus, thereby enhancing its potential as a therapeutic supplement.

### 3.3. Network Pharmacology Analysis of 1,8-CH Antidepressant

**Figure 4:**
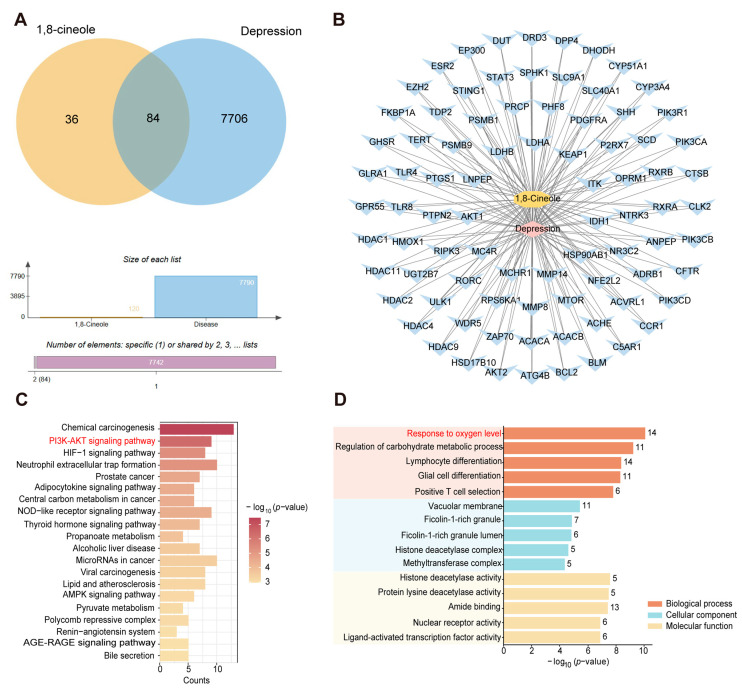
Network pharmacologic analysis of 1,8-CH antidepressant. (**A**) Shared genes between 1,8-CH and depression, identified using Venn diagrams. (**B**) Active compound-target network of 1,8-CH. (**C**) KEGG enrichment analysis. (**D**) GO enrichment analysis.

Through the analysis of the TCMSP database, PubChem, and DrugBank, a total of 120 targets associated with 1,8-CH were identified. Additionally, 7790 targets related to depression were retrieved from disease-specific databases. Among these, 84 targets showed overlap (Figure 4A). PPI network analysis identified key targets, including STAT3, PIK3R1, PIK3CA, PIK3CB, PIK3CD, and AKT1 (Figure S1). The drug–component–disease–target network analysis revealed that 1,8-CH demonstrates a high degree of bioactivity, underscoring its strong potential for therapeutic efficacy as a health supplement (Figure 4B). KEGG pathway analysis indicated that 1,8-CH’s potential benefits in supporting mental well-being could be attributed to its influence on several key pathways, including chemical carcinogenesis, PI3K-AKT signaling, HIF-1 signaling, neutrophil extracellular trap formation, adipocytokine signaling, central carbon metabolism in cancer, NOD-like receptor signaling, and thyroid hormone signaling (Figure 4C). GO analysis indicated that 1,8-CH may be involved in critical biological processes, including the response to oxygen levels, regulation of carbohydrate metabolism, differentiation of lymphocytes and glial cells, and positive selection of T-cells (Figure 4D). Key molecular functions include histone deacetylase activity, protein lysine deacetylase activity, amide binding, nuclear receptor activity, and ligand-activated transcription factor activity. Based on these findings and the current scientific literature [25,26], we propose that 1,8-CH may modulate PI3K-AKT-induced oxidative stress, offering a promising natural strategy for the prevention and management of depression.

**Figure 5:**
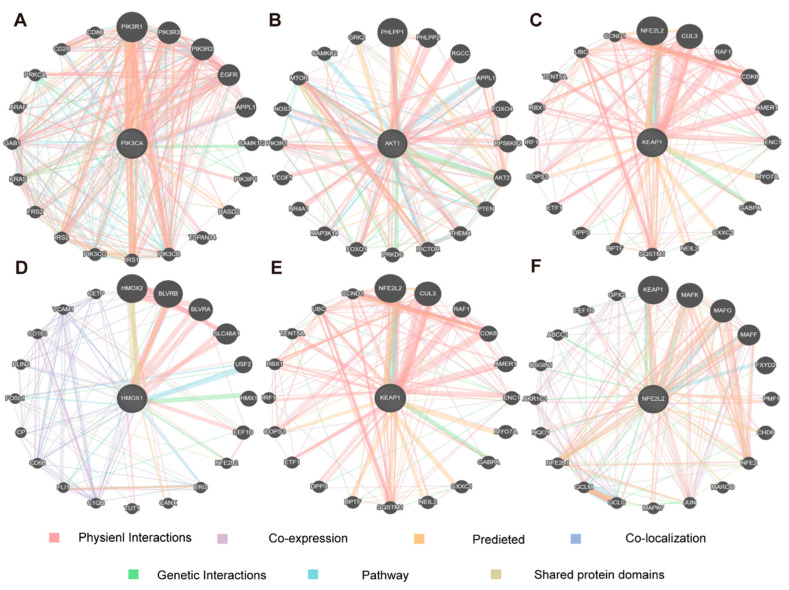
Functional network analysis of key hub targets in the PI3K/Akt/Nrf2 pathway via GeneMANIA. (**A**–**F**) Functional association network of key hub targets in the PI3K/Akt/Nrf2 signaling pathway.

This study employed a functional association network analysis to investigate key hub targets within the PI3K/Akt/Nrf2 signaling pathway using the GeneMANIA tool. This study identified key genes, including PIK3CA, AKT1, Keap1, Hmox1, and Nfe2L2, and mapped their interactions using data from gene co-expression, co-regulation, functional similarity, and protein–protein interactions (Figure 5A–F). The analysis revealed that these genes not only exhibit strong interactions within the PI3K/Akt/Nrf2 pathway but also regulate critical biological processes, including the oxidative stress response, inflammation, and neuroinflammation, which are closely linked to the pathophysiology of depression [27,28]. Notably, hub genes such as Nrf2, AKT1, and PIK3CA are involved in key processes critical for neuronal survival, synaptic plasticity, and mood regulation, indicating their potential role in both the onset and progression of depressive disorders [29,30]. Based on these findings, this study further confirms that 1,8-CH may exert antidepressant effects through the modulation of the PI3K/Akt/Nrf2 pathway.

### 3.4. 1,8-CH Activates the PI3K-Akt Signaling Pathway in the Hippocampus of CUMS-Induced Mice

**Figure 6:**
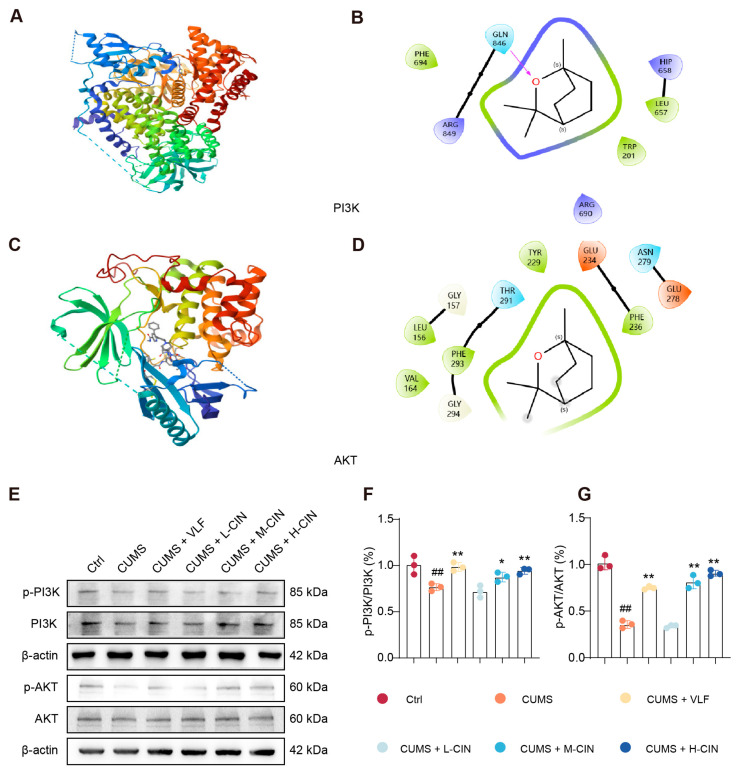
1,8-CH activates the PI3K-Akt signaling pathway in the hippocampus of CUMS-induced mice. (**A**–**D**) Molecular docking of 1,8-CH with proteins associated with the PI3K-AKT signaling pathway. (**E**–**G**) Western blot and quantitative analysis on PI3K-AKT pathway in the hippocampus of CUMS-induced mice. L-CIN, M-CIN, and H-CIN correspond to low, medium, and high doses of 1,8-CH, respectively. Data are expressed as mean ± SD (n = 3). ^#^ *p* < 0.05, ^##^ *p* < 0.01 compared with control group; * *p* < 0.05, ** *p* < 0.01 compared with CUMS group.

Molecular docking analysis revealed that 1,8-CH exhibits a strong binding affinity for phosphoinositide 3-kinase (PI3K) (ΔG = −9.8 kcal/mol) and protein kinase B (AKT) (ΔG = −8.4 kcal/mol), both of which are key proteins of the PI3K-AKT signaling pathway (Figure 6A–D). These results suggest a potential direct interaction between 1,8-CH and these critical signaling proteins, which may contribute to its regulatory effects on the pathway. Given that phosphorylation status is a critical determinant of protein activity, this study quantified the ratios of p-PI3K/PI3K and p-AKT/AKT. The results demonstrated a significant decrease in the phosphorylation levels of both PI3K and AKT in CUMS-exposed mice, indicating the suppression of the PI3K-AKT signaling pathway. However, treatment with 1,8-CH significantly restored phosphorylation levels, suggesting its potential role in modulating this pathway (Figure 6E–G). Collectively, these findings highlight the ability of 1,8-CH to counteract CUMS-induced inhibition of the PI3K-AKT pathway in the hippocampus, providing further evidence of its neuroprotective effects.

### 3.5. 1,8-CH Activates the Nrf2/Keap1/HO-1 Signaling Pathway in the Hippocampus of CUMS-Induced Mice

**Figure 7:**
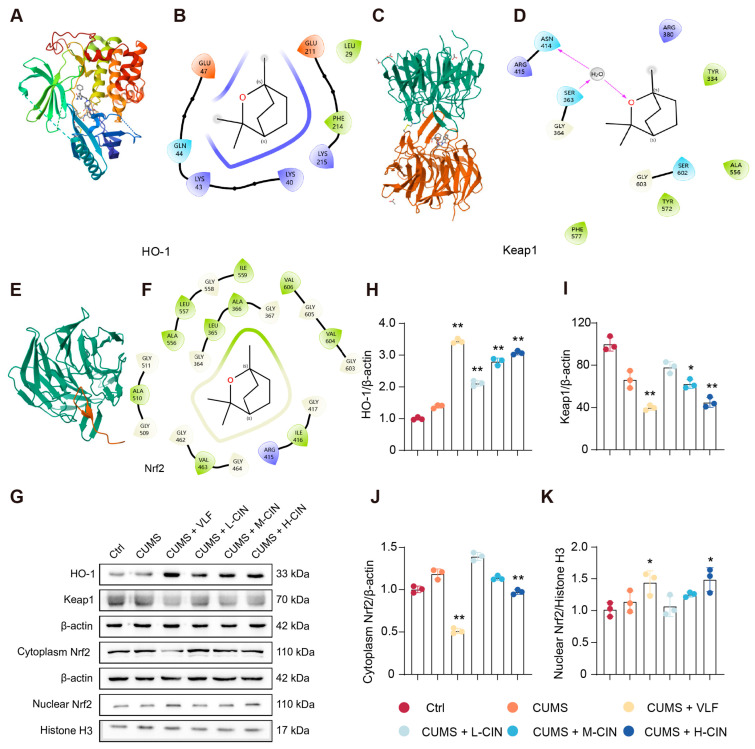
1,8-CH activates the Nrf2/Keap1/HO-1 signaling pathway in the hippocampus of CUMS-induced mice. (**A**–**F**) Molecular docking of 1,8-CH with proteins associated with the Nrf2/Keap1/HO-1 signaling pathway. (**G**–**K**) Western blot and quantitative analysis on Nrf2/Keap1/HO-1 signaling pathway in the hippocampus of CUMS mice. L-CIN, M-CIN, and H-CIN correspond to low, medium, and high doses of 1,8-CH, respectively. Data are expressed as mean ± SD (n = 3). * *p* < 0.05, ** *p* < 0.01 compared with CUMS group.

Molecular docking studies showed that 1,8-CH binds stably to HO-1 (ΔG = −9.3 kcal/mol), Keap-1 (ΔG = −8.3 kcal/mol), and Nrf2 (ΔG = −8.1 kcal/mol), indicating a direct interaction between 1,8-CH and these key molecules in the Nrf2 signaling pathway (Figure 7A–F). Western blot analysis revealed that 1,8-CH treatment significantly improved the Nrf2 signaling pathway in the hippocampus of CUMS mice (Figure 7G). It is important to highlight that there was no significant difference in the expression of the above proteins in the CUMS group compared to the control group. Treatment with 1,8-CH resulted in a significant increase in the expression of HO-1 (Figure 7H), a key antioxidant enzyme regulated by the Nrf2 signaling pathway [31]. This observation suggests that 1,8-CH may effectively augment the cellular antioxidant response, thereby alleviating oxidative stress in the hippocampus of CUMS-induced mice. Furthermore, 1,8-CH treatment resulted in a significant decrease in Keap-1 expression (Figure 7I). Under normal conditions, Keap-1 and Nrf2 exist in a bound state, with Keap-1 acting as a negative regulator of Nrf2. The dissociation of Nrf2 from Keap-1 represents the classical mechanism through which the Nrf2 signaling pathway is activated [32]. This dissociation promotes the stabilization and nuclear translocation of Nrf2, thereby inducing the transcriptional activation of target genes associated with an antioxidant defense and cellular stress response [33,34]. Interestingly, treatment with 1,8-CH resulted in a marked increase in Nrf2 expression in nuclear extracts, alongside a pronounced reduction in its expression in cytoplasmic extracts. This observation suggests that 1,8-CH may promote the nuclear translocation of Nrf2. (Figure 7J,K). Taken together, these results suggest that 1,8-CH modulates the Nrf2 signaling pathway in the hippocampus of CUMS mice, potentially through the upregulation of HO-1, downregulation of Keap-1, and possible facilitation of Nrf2 nuclear translocation. These findings provide robust evidence supporting the potential of 1,8-CH as a dietary supplement for mitigating oxidative stress associated with depression.

### 3.6. Inhibition and Knockdown of Nrf2 Abolishes the Antioxidant Effects of 1,8-CH in CORT-Induced PC12 Cells

**Figure 8:**
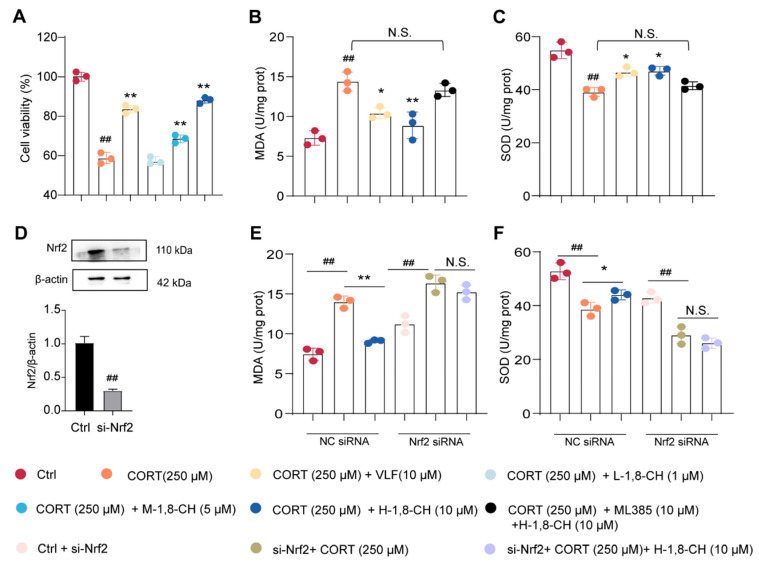
Inhibition and knockdown of Nrf2 abolishes the antioxidant effects of 1,8-CH in CORT-induced PC12 cells. (**A**) The survival rate of PC12 cells was detected by MTT. (**B**) Measurement of MDA levels using an assay kit. (**C**) Measurement of SOD levels using an assay kit. (**D**) The expression of TLR4 protein in si-Nrf2 transfected PC12 cells. (**E**) Measurement of MDA levels using an assay kit. (**F**) Measurement of SOD levels using an assay kit. L-CIN, M-CIN, and H-CIN correspond to low, medium, and high doses of 1,8-CH, respectively. Data are expressed as mean ± SD (n = 3). ^#^ *p* < 0.05, ^##^ *p* < 0.01 compared with control group; * *p* < 0.05, ** *p* < 0.01 compared with CUMS group. N.S. denotes no statistically significant difference between the two groups (*p* > 0.05).

CORT-induced PC12 cells are widely used as an in vitro model to study depression [35]. This study showed that exposure of PC12 cells to 250 µM CORT for 24 h induced significant cellular viability impairment (Figure 8A), which was notably ameliorated by 1,8-CH treatment for 24 h. Quantitative analysis revealed that CORT administration caused marked dysregulation in oxidative stress markers, evidenced by MDA levels and SOD activity (Figure 8B,C). Notably, a 24 h intervention with 1,8-CH effectively restored both MDA and SOD parameters to near-baseline levels, suggesting its potent antioxidant properties. To elucidate the underlying antioxidant mechanisms, the role of 1,8-CH was further investigated using the Nrf2 inhibitor ML385 (10 µM), which was co-administered with CORT. Notably, the protective effects of 1,8-CH were completely abrogated by ML385, highlighting the pivotal role of Nrf2 in mediating its antioxidative properties. These findings were further substantiated by siRNA knockdown experiments (Figure 8D–F), in which Nrf2 expression was silenced for 60 h, confirming its essential role in the protective effects of 1,8-CH against CORT-induced oxidative damage. These findings underscore the pivotal role of Nrf2 as a key regulatory target for 1,8-CH in mitigating CORT-induced oxidative stress in the PC12 cells depression model.

## 4. Discussion

**Figure 9:**
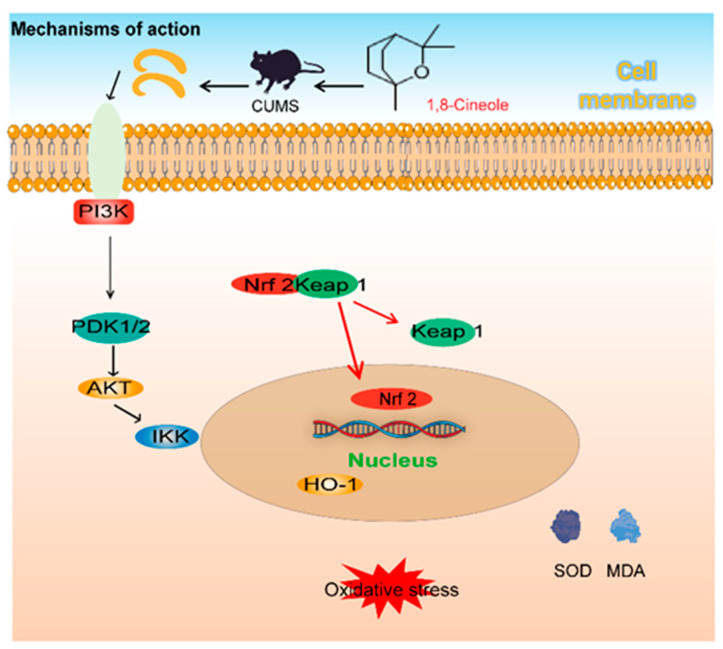
A summary of the mechanisms underlying the antioxidant effects of 1,8-CH in CUMS-induced mice.

This study explores the neuroprotective effects of 1,8-CH as a health supplement in reducing hippocampal oxidative stress in a CUMS mice model of depression (Figure 9). CUMS exposure caused oxidative stress, characterized by elevated MDA levels, reduced antioxidant activity, and neuronal damage. Treatment with 1,8-CH reversed these effects by decreasing MDA, restoring antioxidant enzymes, and reducing ROS accumulation, while improving neuronal integrity. Network pharmacology analysis identified the PI3K/Akt/Nrf2 signaling pathway as a key mediator of 1,8-CH. Western blot analysis further validated these findings, revealing that 1,8-CH activates the PI3K/Akt pathway and possibly promotes the nuclear translocation of Nrf2, thereby enhancing its antioxidant effects. In conclusion, this study found that 1,8-CH alleviates CUMS-induced oxidative stress through the PI3K/Akt/Nrf2 pathway, highlighting its potential as a health supplement for depression management.

The administration of 1,8-CH effectively mitigated the oxidative stress induced by CUMS. Specifically, treatment with 1,8-CH significantly decreased MDA levels, restored the activity of antioxidant enzymes, and reduced ROS accumulation in the hippocampal tissue of CUMS mice. These results suggest that 1,8-CH has potent antioxidant properties, which may contribute to the preservation of cellular redox homeostasis and safeguard neuronal integrity under stress conditions. Notably, the restoration of neuronal integrity and the reduction in ROS accumulation further underscore the potential of 1,8-CH as a health supplement for mitigating oxidative damage associated with depression. One of the key findings of this study is the identification of the PI3K/Akt/Nrf2 signaling pathway as a key mediator of the neuroprotective effects of 1,8-CH. Network pharmacology analysis revealed that 1,8-CH may exert its beneficial effects through the modulation of this pathway, which is known to play a key role in cellular defense mechanisms against oxidative stress. Western blot analysis further revealed that 1,8-CH treatment activates the PI3K/Akt signaling pathway and possibly promotes the nuclear translocation of Nrf2 in the hippocampal tissue of CUMS mice, thereby enhancing the antioxidant response. This finding is particularly significant, as the PI3K/Akt/Nrf2 pathway has been implicated in various neuroprotective and anti-inflammatory processes [36], and its activation may offer a potential mechanism for the development of health supplements aimed at managing depression.

This study’s findings further support the potential of 1,8-CH in managing depression through the modulation of the PI3K/AKT signaling pathway, consistent with emerging research on bioactive compounds in natural products and dietary supplements. Various natural compounds, including flavonoids from *Hippophae rhamnoides* L. (seabuckthorn), essential oils from *Paeonia lactiflora Pall*. (Paeonia), and omega-3 fatty acids like docosapentaenoic acid (DPA), have demonstrated antidepressant effects via the activation of the PI3K/AKT pathway. For example, seabuckthorn flavonoids promote neurotrophic effects through the PI3K/AKT and ERK pathways, essential for synaptic plasticity and neurogenesis [37]. Likewise, Paeonia essential oils attenuate oxidative stress and neuronal apoptosis via the PI3K/AKT/Nrf2 pathway, while omega-3 DPA modulates microglial polarization through the BDNF/TrkB-PI3K/AKT axis, contributing to neuronal survival and function [38,39]. Nrf2, a crucial regulator of oxidative stress and inflammation, plays a key role in neuroprotection [40,41]. The dysfunction of Nrf2 accelerates oxidative damage, compromising neuroprotection and resilience against depression. Health supplements with Nrf2-activating properties, such as fish oil (FO), conjugated linoleic acid (CLA), and Lycium barbarum polysaccharide (LBP), have shown promise in improving mood and emotional well-being [42]. FO and CLA supplementation in MRL/MpJ-Faslpr mice restored redox balance and BDNF expression, suggesting their potential to support mental health through enhanced Nrf2-mediated antioxidant defenses. Similarly, LBP has been shown to improve cognitive and emotional health in a murine model of light exposure, protecting neurons from oxidative stress and activating the Nrf2/HO-1 pathway [43]. Sulforaphane (SFN), a potent Nrf2 activator found in broccoli, has also demonstrated mental health benefits by reducing inflammation and suppressing microglial activation, thus alleviating depression-like behaviors [44]. These findings emphasize the health-promoting potential of using natural supplements to target the PI3K/AKT and Nrf2 pathways for managing depression, providing neuroprotective, anti-inflammatory, and synaptic plasticity-enhancing benefits.

In conclusion, this study highlights the potential of 1,8-CH as a novel functional supplement for supporting mental well-being by targeting oxidative stress via the PI3K/Akt/Nrf2 pathway. Our findings suggest that 1,8-CH may offer a promising nutritional strategy for mitigating oxidative damage associated with depression and promoting neuronal health. However, several key areas warrant further investigation to fully understand its therapeutic potential and the feasibility of its application in health supplements. Firstly, owing to the relatively low bioavailability of 1,8-CH, its full health potential may remain underutilized without the adoption of advanced delivery systems [45]. To optimize its efficacy, strategies such as nanoparticle-based formulations, liposomal encapsulation, or other targeted delivery approaches will be critical for enhancing its absorption, stability, and sustained release. Secondly, a comprehensive assessment of 1,8-CH’s safety profile, including potential toxicity, is imperative. Although promising results have been reported concerning its efficacy and low toxicity [46], long-term safety studies and dose–response evaluations are essential to determine the optimal therapeutic window for its application in mental health management [47]. Lastly, further preclinical studies should explore the broader effects of 1,8-CH, focusing on its impact on behavioral outcomes, interactions with other health supplements or antidepressant treatments, and modulation of additional molecular pathways involved in neuroinflammation and neuroprotection. Investigating its effects across various models of depression and stress, as well as its potential synergistic interactions with other bioactive compounds, could provide valuable insights into its therapeutic potential and versatility as a health supplement. Collectively, these future research directions will be crucial in advancing 1,8-CH toward clinical application, ensuring its efficacy, safety, and therapeutic potential as a health supplement for the prevention and management of depression.

## 5. Conclusions

In summary, this study demonstrates that 1,8-CH protects against hippocampal oxidative stress in a CUMS-induced depression model. It reduces ROS production, restores antioxidant activity, and improves neuronal structure through the PI3K/Akt/Nrf2 signaling pathway. These findings suggest 1,8-CH as a potential supplement for mental well-being and depression management.

## Data Availability

Data are contained within the article.

## Supplementary Materials

The following supporting information can be downloaded at: https://www.mdpi.com/article/10.3390/nu17061027/s1, Figure S1: PPI network construction; Figure S2: (a) PI3K; (b) AKT; Figure S3: (a) HO-1; Keap; (b) Nrf2; Figure S4: Nrf2.
