# Supplementary material for: 1,8-Cineole Alleviates Hippocampal Oxidative Stress in CUMS Mice via the PI3K/Akt/Nrf2 Pathway
Source: Nutrients. 2025 Mar 14;17(6):1027. doi: 10.3390/nu17061027 (PMC11946047; doi:10.3390/nu17061027)

Figure S1. PPI network construction.

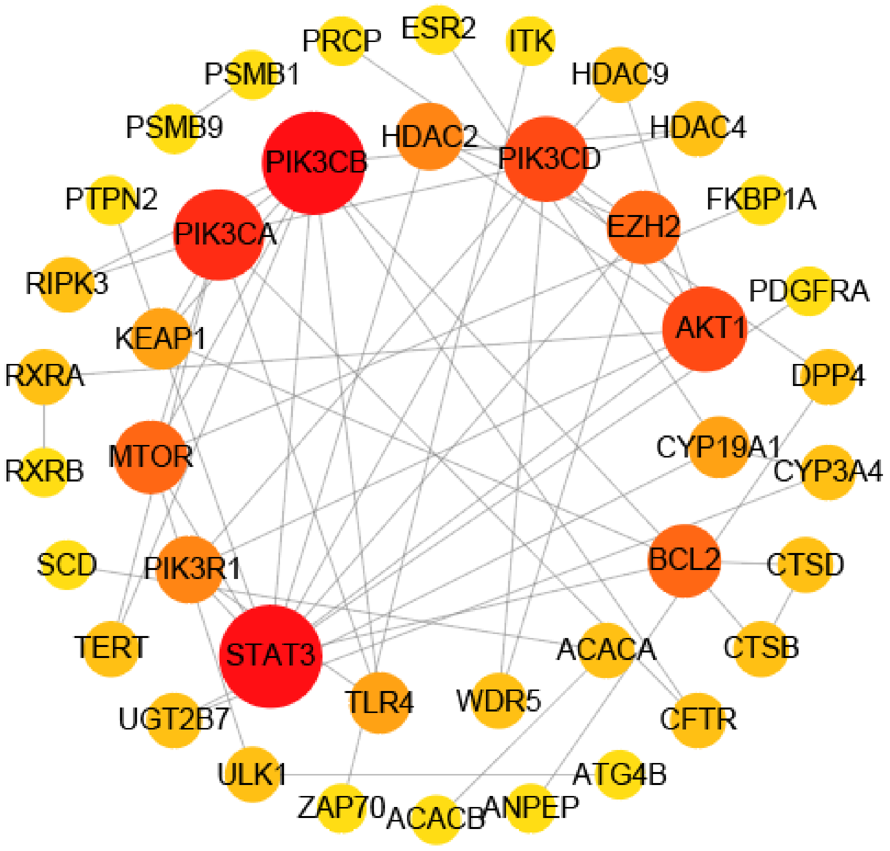

Figure S2. (a) PI3K

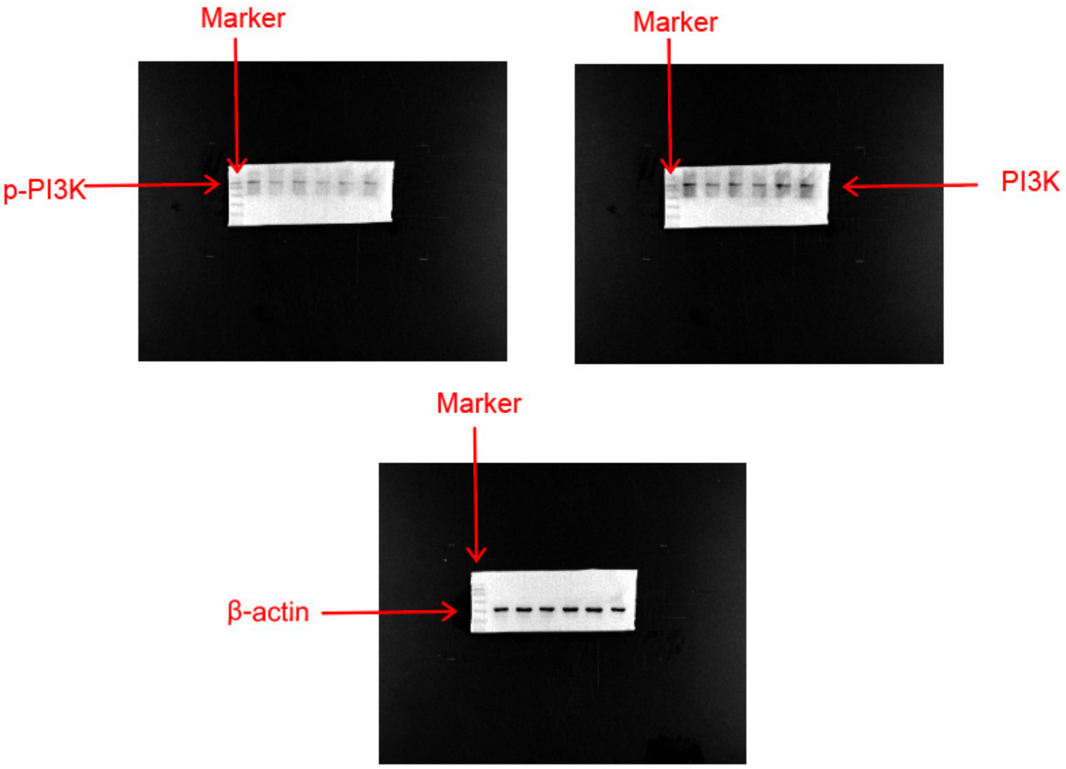

Figure S2. (b) AKT

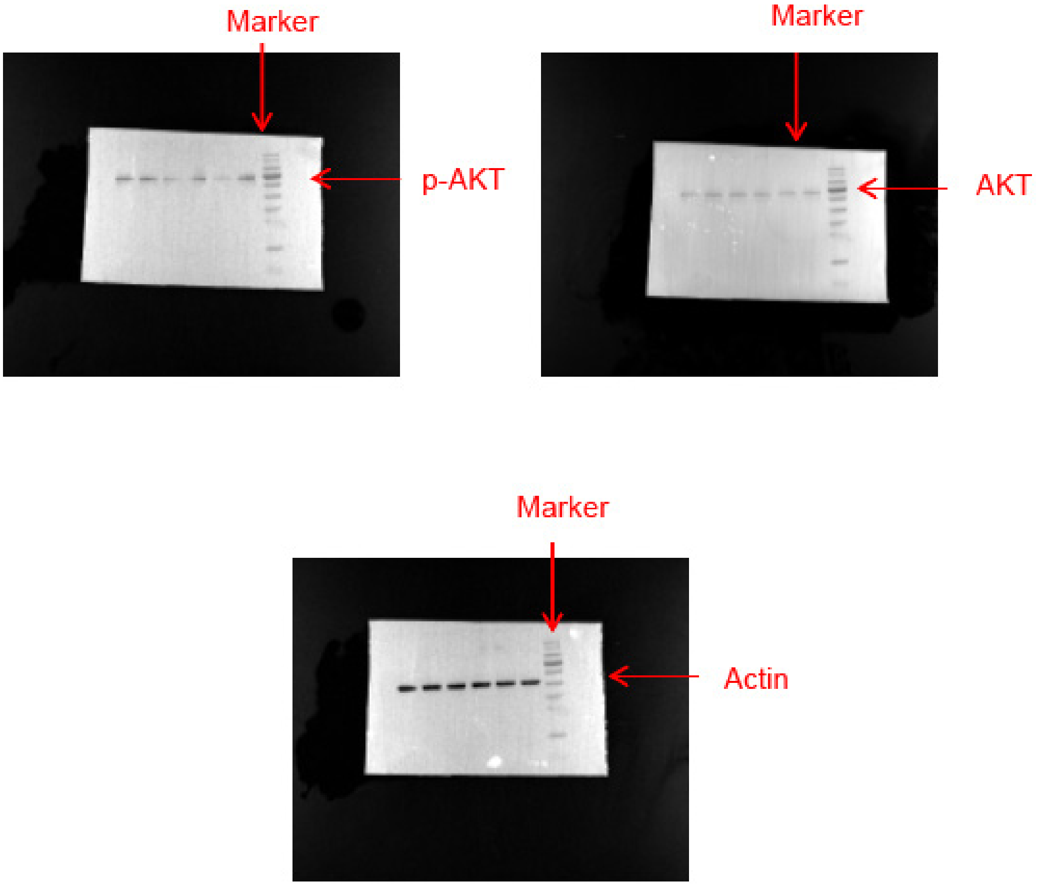

Figure S3. (a) HO-1；Keap

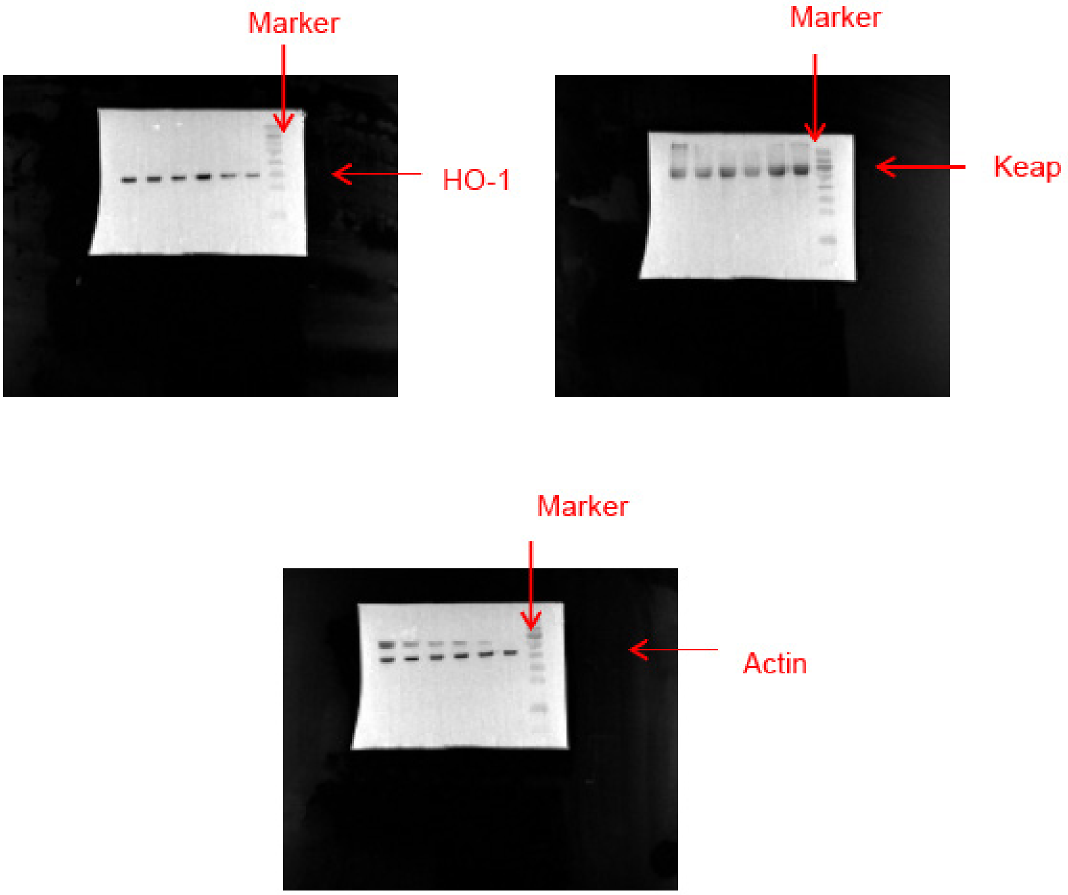

Figure S3. (b) Nrf2

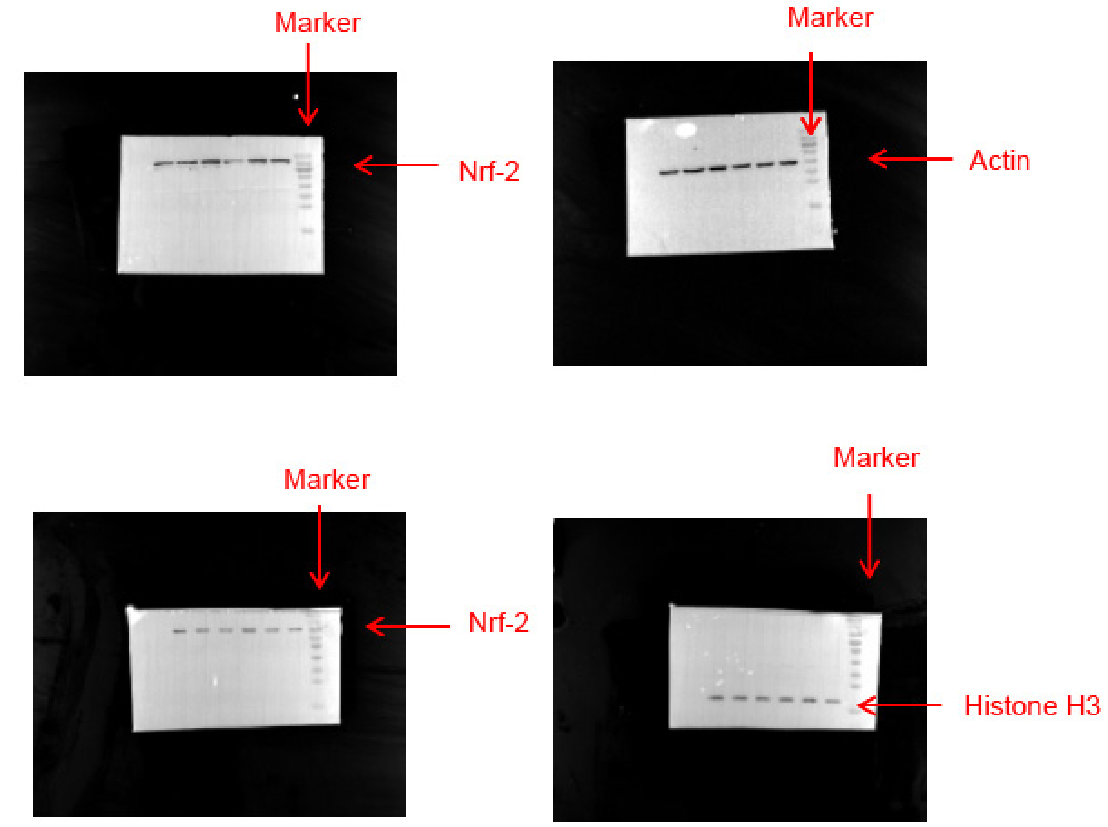

Figure S4. Nrf2

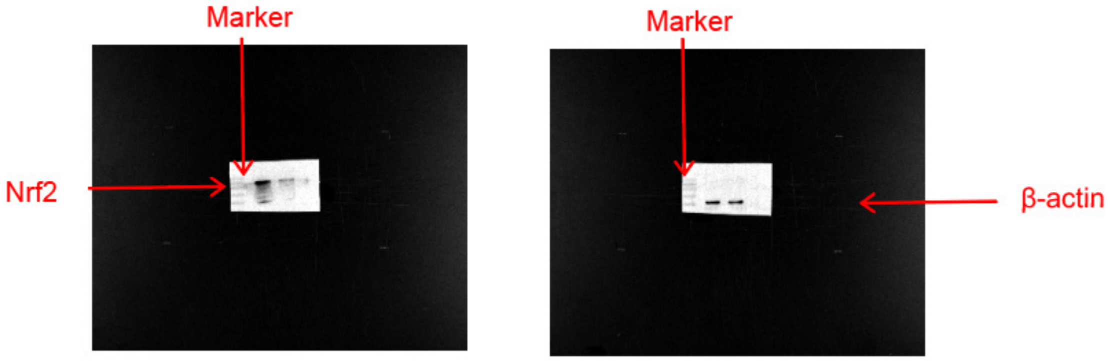

Supplement: Supplementary file 1 [file nutrients-17-01027-s001.zip › nutrients-3490976-supplementary.pdf]
